# Evaluation of the Spatiotemporal Epidemiological Modeler (STEM) during the recent COVID-19 pandemic

**DOI:** 10.1140/epjp/s13360-021-02004-8

**Published:** 2021-10-26

**Authors:** F. Baldassi, F. D’Amico, A. Malizia, P. Gaudio

**Affiliations:** 1 Italian Joint NBC Defence School, Italian Ministry of Defence (MoD), Rieti, Italy; 2 Italian Army Logistic Command - Technical Command, Italian Ministry of Defence (MoD), Rome, Italy; 3 Department of Biomedicine and Prevention, University of Rome “Tor Vergata”, Rome, Italy; 4 Department of Industrial Engineering, University of Rome “Tor Vergata”, Rome, Italy

## Abstract

In early December 2019, some people in China were diagnosed with an unknown pneumonia in Wuhan, in the Hubei province. The responsible of the outbreak was identified in a novel human-infecting coronavirus which differs both from severe acute respiratory syndrome coronavirus and from Middle East respiratory syndrome coronavirus. The new coronavirus, officially named severe acute respiratory syndrome coronavirus 2 by the International Committee on Taxonomy of Viruses, has spread worldwide within few weeks. Only two vaccines have been approved by regulatory agencies and some others are under development. Moreover, effective treatments have not been yet identified or developed even if some potential molecules are under investigation. In a pandemic outbreak, when treatments are not available, the only method that contribute to reduce the virus spreading is the adoption of social distancing measures, like quarantine and isolation. With the intention of better managing emergencies like this, which are a great public health threat, it is important to dispose of predictive epidemiological tools that can help to understand both the virus spreading in terms of people infected, hospitalized, dead and recovered and the effectiveness of containment measures.

## Introduction

In early December 2019 some people in China were diagnosed with an unknown pneumonia in Wuhan, in the Hubei province. The responsible of the outbreak was identified in a novel human-infecting Coronavirus [1, 2] which differs both from severe acute respiratory syndrome coronavirus (SARS-CoV) and from Middle East respiratory syndrome coronavirus (MERS-CoV) [3, 4]. The new Coronavirus, officially named severe acute respiratory syndrome coronavirus 2 (SARS-CoV-2) by the International Committee on Taxonomy of Viruses, has spread worldwide within few weeks. The disease, known as coronavirus disease 2019 (COVID-19), has been supposed to be a zoonotic disease and the person-to-person transmission mainly occurs by direct contact or through droplets spread by coughing or sneezing from an infected individual [5]. It is probably associated with a large seafood and wet animal market in Wuhan City, where live animals are routinely sold, and investigations are ongoing to determine the origins of the infection [6].

The outbreak was declared a Public Health Emergency of International Concern by the World Health Organization on 30th January 2020 and characterized as a pandemic on 11^th^ March. As of December 2020, there have been over 75 million cases and over 1.6 million deaths worldwide since the start of the pandemic [7]. Only two vaccines have been currently approved by regulatory agencies and some others are under development. Moreover, effective treatments have not been yet identified or developed even if some potential molecules are under investigation.

In order to better manage emergencies like this, which are a great public health threat, it is important to implement epidemiological tools that can help to understand both the virus spreading in terms of people infected, dead and recovered and the effectiveness of containment measures.

Here, we use the Spatiotemporal Epidemiological Modeler (STEM release 4.0.1), a Java-based software projected and developed under the umbrella of the Eclipse foundation [8] to understand if this predictive epidemiological tool could be useful to the decision makers in order to reduce the impact of the epidemic. The software has already been proved to be reliable demonstrating to be well-suited to modelling previous Ebola haemorrhagic fever (EHF) epidemics [9] and it has been tested on three real cases of outbreaks: Uganda (2000) [10, 11], Gabon (2001) [12], and Guinea (2014) [13–15]. Moreover, STEM has been previously tested and applied to a hypothetical bioterrorist scenario in order to understand if this tool is able to predict and reduce the impact of this kind of threat; additionally, to comprehend how such tool can support decision makers and policymakers to reduce the spreading of a possible outbreak due to a terrorist attack [16].

## Materials and methods

### Spatiotemporal epidemiological modeler (STEM)

The STEM is an open-source software Java-based, developed under the umbrella of the Eclipse foundation [8]. STEM allows users to create spatial and temporal models of emerging infectious diseases. It was designed to help developers, researchers and users to plug in their choice of models. The user can implement a large number of existing compartment models, e.g. Susceptible/Infectious (SI), Susceptible/Infectious/Recovered (SIR) and Susceptible/Exposed/Infectious/Recovered (SEIR) models pre-coded with both deterministic and stochastic engines, and a new model-building framework that allows users to rapidly extend existing models or to create entirely new models. These models could aid in understanding, and potentially preventing, the spread of a disease.

The STEM application has built-in Geographical Information System (GIS) data for almost every country in the world. Data about country borders, populations, shared borders (neighbours), interstate highways, state highways, and airports can be implemented into the code. This data come from various public sources.

STEM treats the world as a graph within a modular and hierarchical modelling structure. From the bottom to the top, this structure has three basic levels: graphs, models, and scenarios. We refer to STEM tutorial (https://wiki.eclipse.org/Tutorials_for_Developers) for in-depth examination of all the software functions.

Interventions are used in STEM to control some aspect of a disease outbreak, down to regional level if desired. Examples include initiating a vaccination program, isolating infected individuals, implementing social distancing, evacuation of a region, shutting down air transportation (for a county, state or a whole country), closing a road or preventing mixing of infected individuals across borders.

STEM uses triggers, predicates and modifiers to implement interventions. A trigger contains predicate which, when satisfied, invokes one or more modifiers that changes some aspect of a running simulation [8].

### COVID-19 proposed model

In the classical SEIR model, the population is divided in four groups named *S* (susceptible), *E* (exposed), *I* (infectious) and *R* (recovered) [8, 17]. Thus, *N* = *S* + *E* + *I* + *R* refers to the total number of people. The basic hypothesis of the SEIR model is that all the individuals in the model will have the four roles as time goes on. The SEIR model has some limitations for the real situations, but it provides a basic model for the research of different kinds of epidemic.

Starting from the basic SEIR model, we proposed a new model specified by the following equations:

1 $$ \left\{ {\begin{array}{*{20}l} {\frac{{{\text{d}}S\left( t \right)}}{{{\text{d}}t}} = - \frac{S\left( t \right)}{N} \left( {\beta 1I\left( t \right) + \beta 2I_{{\text{i}}} \left( t \right)} \right) + \rho 1Q\left( t \right) - \rho 2S\left( t \right) + \alpha R\left( t \right)} \hfill \\ {\frac{{{\text{d}}E\left( t \right)}}{{{\text{d}}t}} = \frac{S\left( t \right)}{N} \left( {\beta 1I\left( t \right) + \beta 2I_{{\text{i}}} \left( t \right)} \right) - \theta 1E\left( t \right) - \theta 2E\left( t \right)} \hfill \\ {\frac{I\left( t \right)}{{{\text{d}}t}} = \theta 1E\left( t \right) - \gamma 1I\left( t \right)} \hfill \\ {\frac{Ii\left( t \right)}{{{\text{d}}t}} = \theta 2E\left( t \right) - \gamma 2I_{{\text{i}}} \left( t \right) - \varphi I_{{\text{i}}} \left( t \right) + \varepsilon Q\left( t \right)} \hfill \\ {\frac{R\left( t \right)}{{{\text{d}}t}} = \gamma 1I\left( t \right) + \gamma 2I_{{\text{i}}} \left( t \right) + \omega H\left( t \right) - \alpha R\left( t \right)} \hfill \\ {\frac{H\left( t \right)}{{{\text{d}}t}} = \varphi I_{{\text{i}}} \left( t \right) - \omega H\left( t \right)} \hfill \\ {\frac{Q\left( t \right)}{{{\text{d}}t}} = \rho 2\left( t \right) - \varepsilon Q\left( t \right) - \rho 1Q\left( t \right)} \hfill \\ {\frac{{{\text{Deaths}}\left( t \right)}}{{{\text{d}}t}} = \delta 1I\left( t \right) + \delta 2I_{{\text{i}}} \left( t \right) + \delta 3H\left( t \right) - \alpha R\left( t \right)} \hfill \\ \end{array} } \right. $$

where S, E, I, I_i_, R, H, Q and Deaths are the system variables. The descriptions of these variables are presented in Table 1.

**Table 1:** Description of the model variables

Variable	Description
*S*	Susceptible class
*E*	Exposed
*I*	Infectious without intervention
*I*_i_	Infectious with intervention
*R*	Recovered
*Q*	Quarantined
*H*	Hospitalized
Deaths	Disease deaths

**Fig. 1:**
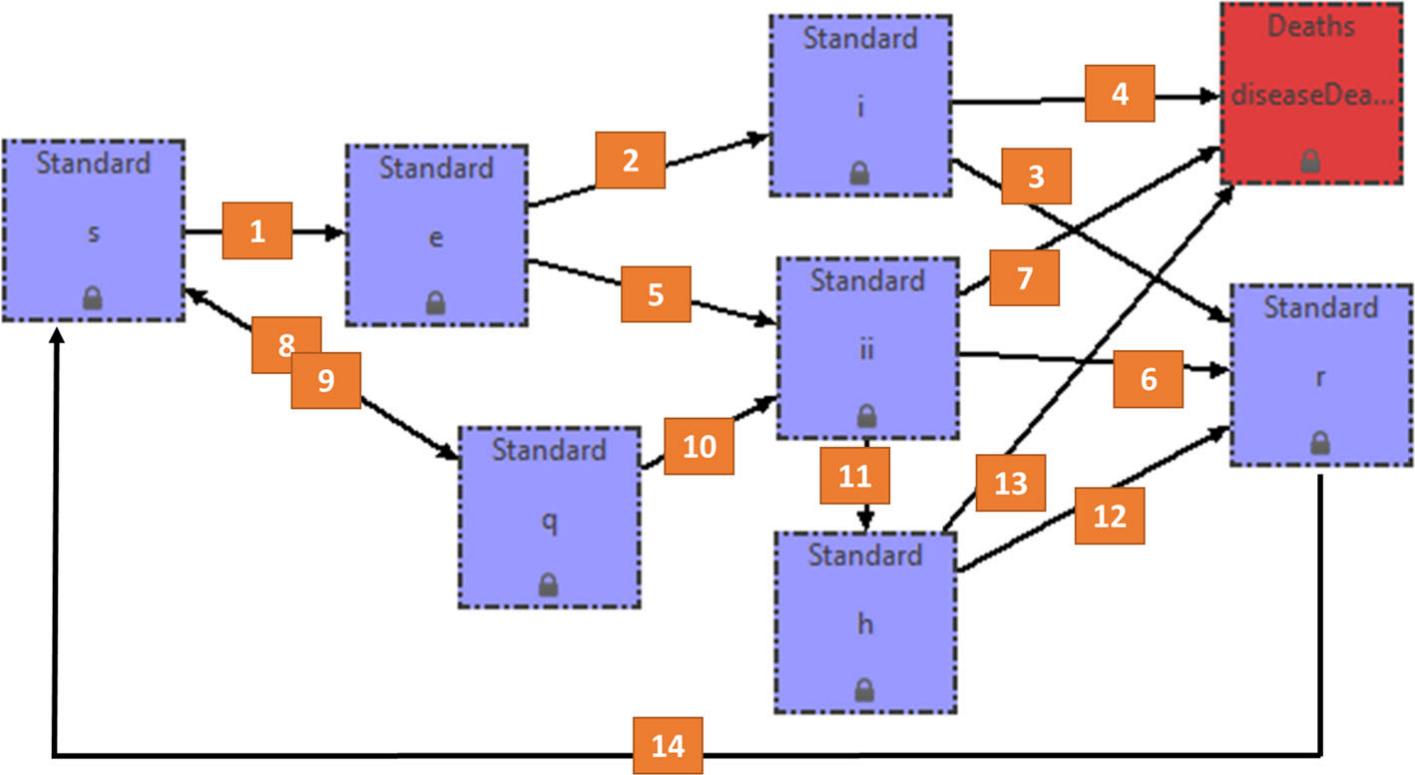
Proposed COVID-19 model. STEM screenshot of the “Generator model” function. The blue and red boxes are the model variables (in minus character). The numbered orange squares are the rates from one box to another, as follows: 1. The contact and infection rate of transmission per contact (*S*/*N(β*_1_*I* + *β*_*2*_*I*_*i*_)); 2. Transition rate of exposed individuals to the infected class (*I*) (*θ*_1_*E*); 3. Recovery rate of symptomatic infected individuals (*I*) to recovered (*γ*_1_*I*); 4. Disease induced death rate due to infectious class (*I*) (*δ*_1_*I*); 5. Transition rate of exposed individuals to the infected class (I_i_) (*θ*_2_*E*); 6. Recovery rate of symptomatic infected individuals (*I*_i_) to recovered (*γ*_2_I_*i*_); 7. Disease induced death rate due to infectious class (*I*_i_) (*δ*_2_*I*_*i*_); 8. and 9. Transition rate of quarantined exposed between the quarantined infected class and the wider community (*ρ*_1_*Q* and *ρ*_2_*S*); 10. Rate of the quarantined class to the recovered class (*εQ*); 11. Rate of infectious with symptoms to hospitalized (*φ*I_*i*_); 12. Recovered rate of quarantined infected individuals (*ωH*); 13. Disease induced death rate due to hospitalized class (*δ*_3_*H*); 14. Temporary immunity rate (*αR*)

The model parameters are illustrated in Table 2, while the relationship between different variables is shown in Fig. 1. In this model, the infectious class is divided into two parts, *I* and *I*_i_. Meanwhile, we consider the quarantined class (*Q*) and hospitalized class (*H*) in the model according to the real situation.

**Table 2:** Description of the model parameters

Parameters	Description
*α*	Temporary immunity rate
*β*_1_, *β*_2_	The contact and infection rate of transmission per contact from infected class
*θ*_1_, *θ*_2_	Transition rate of exposed individuals to the infected class
*γ*_1_, *γ*_2_	Recovery rate of symptomatic infected individuals to recovered
*ε*	Rate of the quarantined class to the recovered class
*φ*	Rate of infected people with symptoms that require hospitalization
*ω*	Recovered rate of quarantined infected individuals
*ρ*_1_, *ρ*_2_	Transition rate of quarantined exposed between the quarantined infected class and the wider community
*δ*_1_, *δ*_2_	Mortality rate of symptomatic infected individuals to deaths
*δ*_3_	Mortality rate of hospitalized class

As shown in Fig. 1, two main channels have been considered in the proposed model. The first one goes to *S* + *E* + *I* + *R*, and the second channel goes to *S* + *Q* + *I*_i_ + *H* + *R*. The first case shows the natural process of the epidemic, and it is a typical SEIR model. The second channel considers possible measures adopted by governments, including quarantine and hospitalization. As a result, the designed model is an improved version of the SEIR model. If there is no quarantine (*ρ*2 = 0), hospital treatment *ω* = 0 and the recovered compartment is immune to the virus (*α* = 0), the model reduces to the classical SEIR model. However, the pandemic has demonstrated that quarantine measures and hospital care were needed almost all over the world. Meanwhile, there is no evidence that the recovered group is immune to the COVID-19. Thus, it is necessary to consider these factors in the model. In this case, *N* = *S* + *E* + *I* + *I*_i_ + *R* + *Q* + *H*, as above, refers to the total number of population and it is in accordance with Eq. (1). Obviously, *N* is not a constant and it varies over time.

The rates of the model proposed are described in details in the caption of Fig. 1.

### Parameters estimation

The actual COVID-19 data from Hubei province have been utilized to estimate the parameters of the proposed SEIR model to fit the real situation. The COVID-19 data were taken from the official website of the Wuhan Municipal Health Commission (http://wjw.wh.gov.cn/).

In order to prevent and control the epidemic, Wuhan government announced to seal off the city from the rest of the world on 23rd January 2020. Later on, other cities in Hubei province adopted the same measure. The COVID-19 pandemic situation of Hubei was relatively stable after 23rd January 2020, so we chose to evaluate the proposed model with the data between January 24th and April 12th.

In the proposed SEIR model N is the total population of Hubei pre-loaded in STEM, and *E* is calculated based on the number of confirmed patients. *I* is an estimated value based on *I*_i_, and the other initial values are originated from the actual data [18]. The initial parameters of the SEIR model were set as follows: *N* = 16 × 10^6^, *E* = 5077, *I* = 7, *I*_i_ = 730, *H* = 658, *R* = 32, and *Q* = 4711.

The model parameters of the proposed model are calculated and estimated by the scientific literature data [18–24], as shown in Table 3.

**Table 3:** Model parameters of the model proposed

Parameter	Value	References
*β*_1_	1.0538 × 10^–1^	[18–24]
*β*_2_	1.0538 × 10^–1^	
*ρ*_1_	2.8133 × 10^–3^	
*ρ*_2_	1.2668 × 10^–1^	
*θ*_1_	9.5000 × 10^–4^	
*θ*_2_	3.5412 × 10^–2^	
*γ*_1_	8.5000 × 10^–3^	
*γ*_2_	1.0037 × 10^–3^	
*ε*	9.4522 × 10^–2^	
*α*	1.2048 × 10^–4^	
*φ*	2.9100 × 10^–1^*, 9.7300 × 10^–2^**, 6.0000 × 10^–2^***	
*ω*	1.0700 × 10^–2^ *, 4.1600 × 10^–2^ **, 6.5000 × 10^–2^***	
*δ*_1_	4.0000 × 10^–3^	
*δ*_2_	5.0350 × 10^–3^	
*δ*_3_	5.0000 × 10^–4^	

*Valid from 24 January to 8 February; **valid from 9 to 19 February; ***valid from 20 February to 11 April

However, there is no accurate statistics of the rate of infected people with symptoms that require hospitalization (*φ*) and the recovered rate of quarantined infected individuals (*ω*). Here, the two parameters are estimated by the literature and the actual data of *R* and *H*.

## Results

The model in Fig. 1 has been developed in STEM using its “Model generator” function and once realized, the parameters in Table 3 have been loaded in the model. The initial values of the model are reported in paragraph 2.3.

The study has been divided in two main parts. In the first one, the proposed model has been calibrated and adjusted with real data taking into account two different stages of COVID-19 epidemic. In the second one, the model has been used to evaluate STEM capabilities and characteristics to reduce the impact of an outbreak and consequently to help decision makers and health care workers for contrasting emergency like COVID-19.

**Fig. 2:**
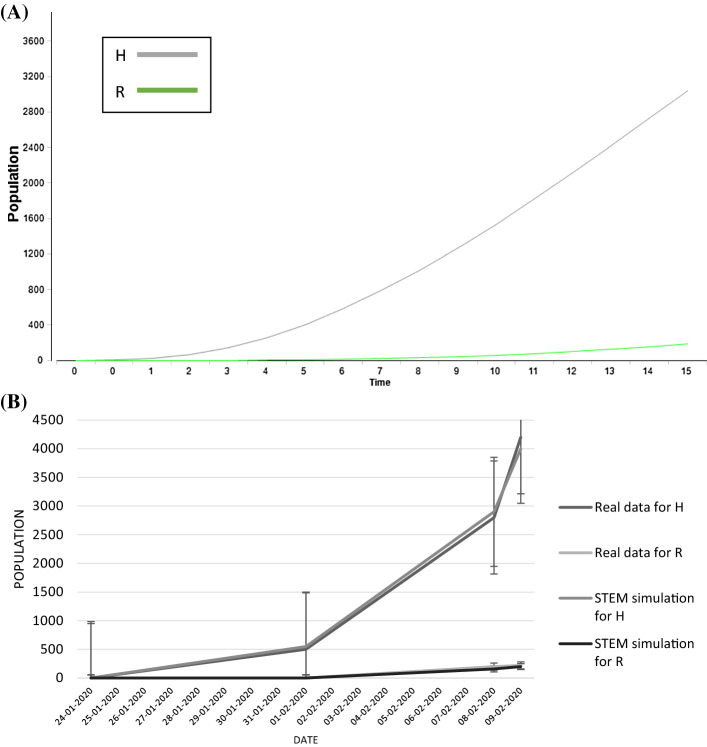
Outbreak stage simulation and comparison graph. The legend is on the right and the errors considered are standard errors. **A** STEM simulation due to the application of the model proposed in the outbreak stage: in green the recovered people and in grey the hospitalized people. Time: days. **B** The graph reported the comparison between the STEM simulations and the real data taking into the account the *H* and *R* classes (colour legend on the right). Date: days of month

**Fig. 3:**
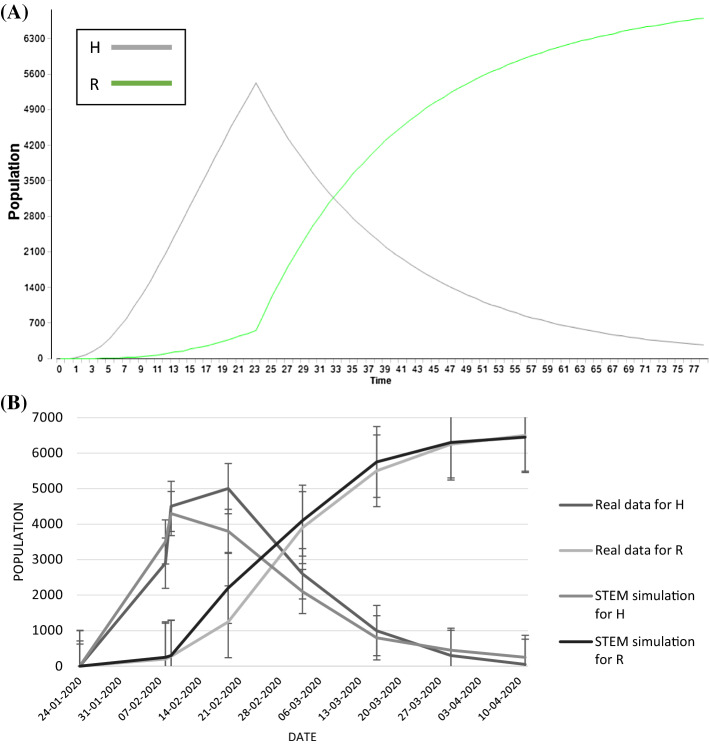
Outbreak and inhibition stages STEM simulation and comparison graph. The legend is on the right and the errors considered are standard errors. **A** STEM simulation due to the application of the model proposed in the outbreak and inhibition stages together: in green the recovered people and in grey the hospitalized people. Time: days. **B** The graph reported the comparison between the STEM simulations and the real data taking into the account the *H* and *R* classes (colour legend on the right). Date: days of month

Starting with the first part of the study, according to the references [18–20], the COVID-19 epidemic situation in Hubei is divided into two stages: the outbreak stage (the first 19 days) and the inhibition stage (the 20th day to 11 April 2020). Therefore, the parameters in Table 3 have been fitted in the model in Fig. 1 and the results of the simulations are shown in Fig. 2 and 3. In particular, in Fig. 2A it is possible to see the STEM simulation for outbreak stage in the first 20 days in Hubei province, and in Fig. 2B a graph where there is a comparison between the simulation data and the real data for this stage.

In Fig. 3A, it is possible to see the STEM simulation for inhibition stage in Hubei province, while in Fig. 3B a graph, as already done for the outbreak stage, where it has been compared the simulation data and the real data.

In the second part of this study, once calibrated the proposed model with real data, it has been taken into the consideration the application of the proposed model in STEM with the addition of others countermeasures or preventive actions not specifically medicals: social distancing and wearing Personal Protective Equipment (PPE). Social distancing, also called “physical distancing,” means keeping a safe space between yourself and other people who are not from your household [25]. For wearing PPE means wear face mask, as medical/surgical face masks or N95 respirators.

These countermeasures have been applied 15 days after the beginning of the outbreak in Hubei province (24 January 2020). For the application of these no-medical countermeasures (social distancing and wearing masks) the model parameters have been recalculated and arranged in STEM as a trigger. The choice of what type of parameters takes into the account and its recalculation is based on the references. It has been assumed that the application of these additional preventive actions should reduce the transmission rates (*β*_1_, *β*_2_) of 40% [20, 26–28].

**Fig. 4:**
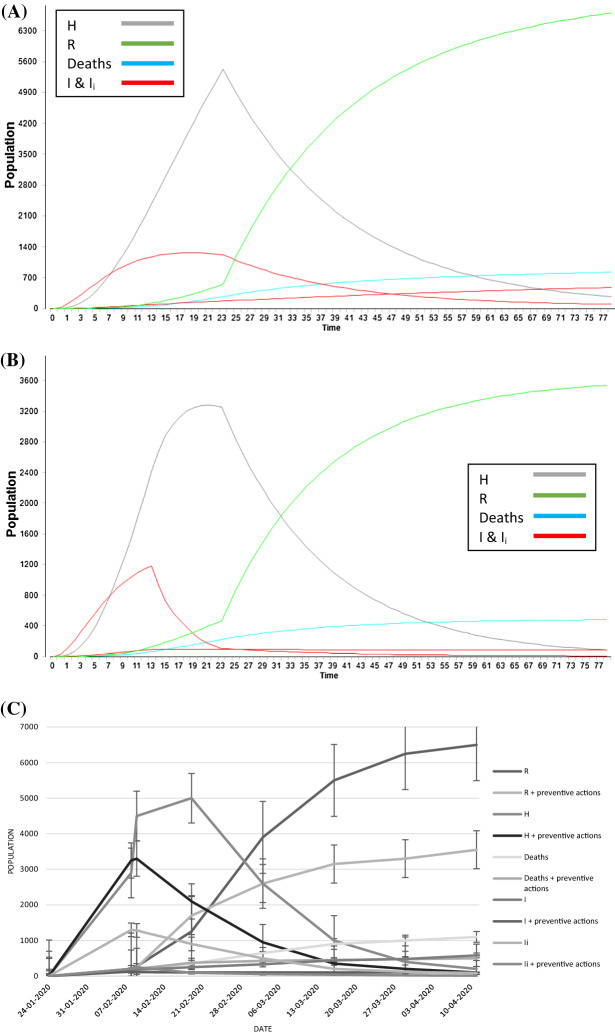
Application of additional countermeasures. The legend is on the right and the errors considered are standard errors. **A** STEM simulation with the model proposed: *H* (grey line), *R* (green line), Deaths (light blue line), *I* and *I*_i_ (red line). Time: days. **B** STEM simulation considering additional non-medical countermeasures (use of PPE and social distancing): *H* (grey line), *R* (green line), Deaths (light blue line), *I* and *I*_i_ (red line). Time: days. **C** The graph reported the comparison between the STEM simulations in *A* and *B* and the real data taking into the account the *H* and *R* classes. Date: days of month

The aim is to understand and study what would have been the impact of further countermeasures in the same scenario considered for standardizing the model. Outcomes are reported in Fig. 4. Particularly, Fig. 4a reports the STEM graph as a result of the simulation obtained with the application of the calibrated model with real data; Fig. 4b shows the STEM simulation graph obtained considering the preventive actions discussed above. Figure 4c displays the comparison of the data from Fig. 4a, b. In these last simulations, also the infected classes (I, I_i_) and deaths class (Deaths) have been analysed.

## Discussion

As the intention of the authors, the approach here developed should be the same that STEM end users could apply in an emergency situation as COVID-19 in order to help decision makers and stakeholders to reduce the impact of infectious disease in the population affected.

The first step has been to elaborate a specific model for COVID-19 taking into account the available scientific literatures data (Fig. 1). Then, the model has been developed using a STEM function called “Model generator”. Once ready, the proposed model has been loaded in a STEM scenario in order to evaluate and calibrate the model with real data. The COVID-19 epidemic situation in Hubei has been divided into two stages: the outbreak phase (the first 19 days) and the inhibition phase (from the 20th day to the end—11 April 2020). As reported in Table 3, the parameters of the system are mainly chosen by two means including the references. For instance, the contact and infection rate parameters are defined according to references [18–24]. In the outbreak stage, according to the actual data of R and H, the φ and the ω are estimated to *φ* = 2.91 × 10^–1^, *ω* = 1.07 × 10^–2^, respectively. After the outbreak stage, due to the continuous assistance from other provinces and other countries, the epidemic in Hubei began to enter the inhibition stage and the estimated φ and ω changed to *φ* = 9.73 × 10^–2^, *ω* = 4.16 × 10^–2^ until 19 February and *φ* = 6 × 10^–2^, ω = 6.5 × 10^–2^ until 11 April 2020, respectively [18, 19].

The estimated and actual trajectories in the two stages are shown in Figs. 2 and 3. In the first stage, although there are some errors between the estimated and the actual numbers, the estimated values well match with the real situation (Fig. 2b). The accuracy is also satisfied in the second stage (Fig. 3b) which shows that the real data are almost the same than the estimated values, and the trend is basically overlapped. It has been decided to focus the analysis on estimation of R and H for two reasons mainly: first, they are the more available and genuine data and second, these classes are important to take into consideration when decision makers and stakeholders have to control and set specific countermeasures against an epidemic.

**Fig. 5:**
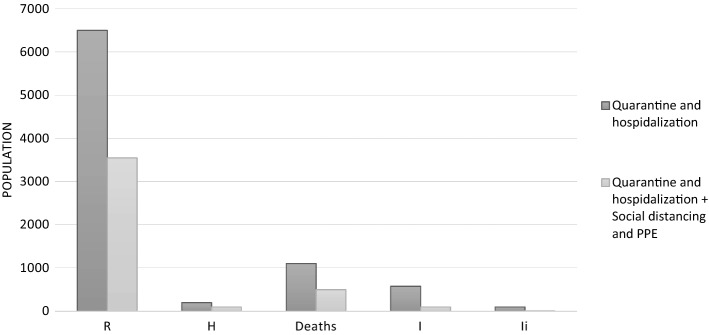
Bar plot of the comparison between data of Fig. 4. In dark grey the outcomes with the application only of quarantine and medical treatment, in light grey the results with the further consideration of others no medical countermeasures, as social distancing and use of PPE

In the second part of the work, once calibrated the proposed model with real data, the intention was to consider the application of the proposed model in STEM with the addition of others countermeasures not specifically medicals, as social distancing (at least 1 m of distance between 2 people) and wearing personal protective equipment (mask). As already underlined above, these countermeasures have been applied 15 days after the beginning of the outbreak in Hubei province. In Fig. 5, the effects of a possible application of preventive countermeasures to contrast the epidemic are shown. In this case, other classes of population as Deaths and Infected people (*I*, *I*_i_) have been taken into account.

As evident, in all considered classes there is a clear reduction of the number of people involved and affected. Specifically, it is possible to estimate the following percentages of reduction: 46% for R class, 50% for H class, 55% for Deaths class and 83% for both infected classes (*I* and *I*_i_).

This work demonstrates how an end user, as an epidemiologist or a public health expert, can use a tool such STEM to evaluate the impact of different strategies in order evaluate the efficacy of some countermeasures before their application. Once a reference model is developed based on available data, it is straightforward to integrate the reference model into the future subject to a range of plausible assumptions. With the application of same interventions, the base model predicts a reduction of more than 50% of people involved in COVID-19 epidemic.

There are several challenges to tackle modelling the effect of specific countermeasures on COVID-19 transmission for a spatially local region such as Hubei province. First of all, the specified COVID-19 model itself is not a perfect representation of the world, as with all models. Secondly, there are challenges in defining the initial condition for our simulation. There is a weakness in the assumption that, for instance, it is slightly impossible determine how many people are infected in reality and consequently how many are exposed.

We demonstrated the usefulness of using an open source tool, as STEM, both to model infectious disease spread and to measure the impact of alternative intervention strategies such as improved no medical countermeasures, as PPE and social distancing. The model used is available to any researcher to use freely, allowing transparency of analysis for peer refinement and critique. As George E. P. Box observed: “essentially, all models are wrong, but some are useful” [29].

Modelling can advise the development of public health policy, but given the uncertainties associated with public health data, it is essential that the assumptions built into such models and the models themselves be fully transparent. Perhaps the greatest strength of STEM is not the use of advanced software technology but the transparency that comes with open source.

## Conclusions

The proposed model has been built using the STEM function “Model generator” and then evaluated in accordance with the reference literature. This model has revealed itself as suitable for the dynamics of the epidemic of COVID-19. Thus, once loaded in STEM function “Scenario designer”, it has been tested and calibrated using two different stages of the epidemic: outbreak stage (no countermeasures), inhibition stage (quarantine and medical treatment/hospitalization). Successively, the fixed model has been applied in a specific scenario in order to study and evaluate the outcomes if both additional no-medical countermeasures were taken and when social distancing and wearing PPE were applied. The STEM simulations analysed the effects of epidemic behaviour change alone and in combination with specific control measures. The provided information can suggest to decision makers, with a credible level of accuracy, how the outbreak would spread and develop in space and time in different phases. As a result, this tool could help to develop (and test) control strategies based on computer simulations.

## Data Availability

This manuscript has associated data in a data repository. [Authors’ comment: The datasets analysed and generated during the current study are available upon reasonable request by contacting the corresponding author.]

## Abbreviations

CDC: US Centers for Disease Control and Prevention
COVID-19: Novel coronavirus diseases
E: Exposed
EHF: Ebola haemorrhagic fever
GIS: Geographical information system
H: Hospitalized
I: Infectious without intervention
I_i_: Infectious with intervention
MERS: Middle east respiratory syndrome
MERS-CoV: Middle east respiratory syndrome coronavirus
PPE: Personal protective equipment
Q: Quarantined
R: Recovered
S: Susceptible class
SARS: Severe acute respiratory syndrome
SARS-CoV: Severe acute respiratory syndrome coronavirus
SARS-CoV-2: Severe acute respiratory syndrome coronavirus 2
SEIR: Susceptible-exposed-infected-recovered
SI: Susceptible/infectious
SIR: Susceptible-infected-recovered
STEM: Spatiotemporal Epidemiological Modeler
WHO: World Health Organization

## Code availability

https://www.eclipse.org/stem/.
